# The cut-off levels of procalcitonin and C-reactive protein and the kinetics of mean platelet volume in preterm neonates with sepsis

**DOI:** 10.1186/s12887-018-1236-2

**Published:** 2018-08-01

**Authors:** C. Aydemir, H. Aydemir, F. Kokturk, C. Kulah, A. G. Mungan

**Affiliations:** 1 Department of Pediatrics, Medical Faculty, Division of Neonatology, Bülent Ecevit University, Zonguldak, Turkey; 2 Department of Infectious Diseases and Clinical Microbiology, Medical Faculty, Bulent Ecevit University, 67600 Zonguldak, Turkey; 3 Department of Biostatistics, Medical Faculty, Bulent Ecevit University, Zonguldak, Turkey; 4 Department of Microbiology, Medical Faculty, Bulent Ecevit University, Zonguldak, Turkey; 5 Department of Biochemistry, Medical Faculty, Bulent Ecevit University, Zonguldak, Turkey

**Keywords:** Neonatal sepsis, Procalcitonin, CRP, MPV

## Abstract

**Background:**

Sepsis is a leading cause of morbidity and mortality among newborns. C-reactive protein (CRP) and procalcitonin (PCT) have some limitations in the diagnosis of preterm neonatal sepsis. In this study, the cut-offs of PCT and CRP, and the efficacy of mean platelet volume (MPV) were investigated.

**Methods:**

We identified key demographic details and compared laboratory values between preterm infants with early onset and late onset neonatal sepsis (EONS/LONS) retrospectively. Blood samples were collected within the first few hours of the onset of clinical sepsis (CRP 1, PCT 1, MPV 1) and were repeated after 24 h (CRP 2, PCT 2, MPV 2). The optimal cut-offs for CRP, PCT and MPV were determined using receiver operating characteristic (ROC) analysis. Furthermore, pairwise comparisons of ROC curves were made to evaluate the performances of these tests.

**Results:**

In EONS, the cut-off of CRP 1 was 2.6 mg/L, the sensitivity, specificity, PPV and NPV were 80.6, 83.0, 67.5 and 90.7%, respectively (*p* < 0.001). At a PCT 1 cut-off of 1.1 ng/mL, the sensitivity, specificity, PPV and NPV were 78.6, 81.2, 64.7 and 89.6%, respectively (*p* < 0.001). The sensitivity, specificity, PPV, and NPV of the CRP 1 cut-off of 3.6 mg/L for LONS were 78.3, 87.4, 74.8, and 89.4%, respectively. At a PCT 1 cut-off of 5.2 ng/mL, the sensitivity, specificity, PPV and NPV were 58.5, 95.5, 86.1, and 82.9% respectively. For proven sepsis, the cut-off of CRP 1 was 7.0 mg/L with a 76.5% sensitivity, 98.2% specificity, 94.9% PPV and 90.5% NPV (*p* < 0.001). At a PCT 1 cut-off of 1.36 ng/mL, the sensitivity, specificity, PPV and NPV were 90.8, 83.4, 70.6 and 94.4%, respectively (*p* < 0.001). In each subgroup, other than EONS, the performances of CRP 1 and PCT 1 measurements were found to be statistically higher than MPV 1. CRP 2 cut-off levels of LONS group and proven sepsis group were found to be lower than the initial values.

**Conclusions:**

Optimal cut-off levels of CRP 1 and PCT 1 may differ in preterm sepsis subgroups. The diagnostic performances of CRP 1 and PCT 1 didn’t differ however, they were more efficacious than MPV.

## Background

Sepsis is a major source of morbidity and mortality in the neonatal population and preterm infants are disproportionally affected [1]. Although positive blood culture is the gold standard used for definitive diagnosis of infection, this test is limited by the length of time it takes to grow microorganisms and potential for interference due to contamination [2, 3]. As clinical signs and symptoms can be very subtle and may mimic non-infectious conditions, there is a need to establish a range of effective biomarkers to aid in prompt decision making [1]. For the rapid identification of microorganisms causing sepsis, novel laboratory methods, such as cytokine and molecular analyses, have been developed; however, it is unlikely that these methods will be useful in the near future because they are not very cost effective [3, 4]. Serum C-reactive protein (CRP) and procalcitonin (PCT) are two of the most thoroughly investigated laboratory markers used to diagnose neonatal sepsis. They have proven to be helpful in the early diagnosis of bacterial invasion; however, they may also be affected by maternal and fetal non-infectious conditions [4]. PCT was suggested to increase physiologically in healthy preterm neonates during the early neonatal period [5, 6]. Gestational age was found to be one of the independent factors that influence the concentration of PCT [6]. Thrombocytopenia has been used as an early but nonspecific marker for sepsis [7, 8]. It has also been associated with prolonged hospitalization and reduced survival rates in previous studies [9–11]. The mean platelet volume (MPV) is another marker that has been available since the 1970s [12]. Although MPV measurements in healthy populations showed an inverse relationship with the platelet count, the clinical meaning of this relationship in neonates with sepsis has not been thoroughly investigated [13]. The objectives of this study were to evaluate the kinetics of MPV measurements, and PCT and CRP levels in preterm newborn sepsis. We also aimed to determine the most appropriate cut-off values for CRP, PCT and MPV using receiver operating characteristic (ROC) curves and identify the diagnostic sensitivity, specificity, positive predictive value (PPV), and negative predictive value (NPV) of each cut-off in early-onset preterm neonatal sepsis (EONS), late-onset preterm neonatal sepsis (LONS), proven and clinical preterm neonatal sepsis. Furthermore, we identified the differences in cut-offs between sepsis subgroups. Because immediate start of antibiotic therapy influences the final outcome in preterm septic neonates, we believe the cut-off levels of these parameters and the performances of these tests are important in the diagnosis of sepsis, especially for prematures.

## Methods

### Patients

Bulent Ecevit Teaching and Research Hospital is a 600-bed tertiary care hospital in Zonguldak, Turkey. The hospital contains a 20-bed neonatology intensive care unit. This unit offers level-3 neonatal intensive care, except for neonatal surgery, for West Blacksea region of Turkey (approximately 450 newborn hospitalizations per year, 60% of them are preterms). Newborns with at least two clinical symptoms and at least two laboratory signs regarding clinical, hemodynamic, tissue perfusion or inflammatory variables (from the Table 1) in presence of a result of suspected or proven infection (positive culture) and for whom there was no other reason to explain these findings other than infection were diagnosed with sepsis. Patients who fulfilled these criteria within 3 days of life were defined as having EONS. If the diagnosis of sepsis was made after 3 days of life, it was defined as LONS [14]. Proven sepsis was defined if the causative microorganism was isolated from the blood. Demographic findings, including prenatal, natal and postnatal histories, maternal age, gestational age, intrapartum maternal fever, presence of preterm and or prolonged rupture of membranes, chorioamnionitis, antibiotic treatment, clinical features, laboratory results and outcomes were recorded. Our retrospective study population consisted of 204 preterm septic newborns with a gestational age between 23 and 36 weeks hospitalized between January 2013 and December 2016. Fifteen newborns who had one of the following conditions were excluded from the study: chromosomal abnormality, major congenital malformation, multi-organ failure as a result of noninfectious conditions, malignancy, perinatal hypoxia, and asphyxia.

**Table 1:** Clinical and laboratory signs for sepsis diagnosis [33]

Clinical signs	Laboratory signs
Modified body temperature: 1-Core temperature greater than 38,5 °C or less than 36 °C and/or 2-Temperature instability	Leucocyte count: 1- < 4000/mm^3^ or 2- > 20,000/mm^3^
Cardiovascular Instability: 1-Bradycardia 2-Tachycardia 3-Rhythm instability: 4-Reduced urinary output (less than 1 ml/kg/h), 5-Hypotension 6-Mottled skin, 7-İmpaired peripheral perfusion	Immature to total neutrophil ratio: ≥0.2
Skin and subcutaneous lesions: 1-petechial rash 2-sclerema	Platelet count < 100,000 /mm^3^
Respiratory instability: 1-Apnoea or 2-Tacypnoea or 3-Requirement of ventilation support	CRP > 15 mg/L or PCT ≥ 2 ng/mL.
Gastrointestinal: 1-Feeding intolerance 2-Poor sucking 3-Abdominal distention	Glucose intolerance confirmed at least 2 times: 1-Hyperglycaemia (blood glucose > 180 mg/dL or 10 mmol/L) or 2-Hypoglycaemia (glycaemia< 45 mg/dL or 2.5 mmol/L)
Nonspesific: 1-Irritability 2-Lethargy 3-Hypotonia	Metabolic acidosis: 1-Base excess (BE) < − 10 mEq/L or 2-Serum lactate > 2 mmol/L

*CRP* C-reactive protein, *PCT* Procalcitonin

### Control group

They had no clinical signs of sepsis. The platelet, MPV measurements, CRP, and PCT levels were obtained from these newborns at the moment of hospitalization. The control group was admitted to the hospital for perinatal conditions other than infection, such as hypoglycemia, indirect hyperbilirubinemia, or intrauterine growth restriction. They did not have treated with an antibiotic regimen. They had no diagnosis of sepsis during the hospitalization period. As a result, 223 infants fulfilled the inclusion criteria for the control group and therefore became the control population for the study.

### Blood sampling, isolation and identification of microorganisms from cultures

Blood samples were obtained within the first few hours of the onset of clinical sepsis. The variables included hemoglobin, hematocrit, platelet count, MPV 1, leucocyte count, CRP 1, PCT 1, and blood culture. Blood sampling was repeated after 24 h including MPV 2, CRP 2, PCT 2. This blood sampling time interval for measurement of these parameters is a part of our hospital’s neonatal sepsis protocol. Peripheral blood cultures were obtained before antibiotic treatment or before switching the antibiotic for clinical sepsis diagnosis. Urine and cerebrospinal fluid were cultured only when clinically indicated. Two positive blood cultures were required to confirm *Staphylococcus epidermidis* sepsis. Blood cultures were not routinely taken from the control preterm patients.

Blood cultures were performed in the BACTEC 9120 blood culture system (Becton Dickinson, USA). Isolates were identified using conventional methods, and when required, the results were confirmed by semi-automated API systems (bioMe’rieux, Marcyl’Etoile, France). Antibiotic susceptibility tests were performed by the Kirby–Bauer disk diffusion method according to the guidelines of the Clinical and Laboratory Standards Institute (CLSI) standards M100-S20–25.

Blood for complete blood counts was obtained via venipuncture, arterial puncture or a central catheter. The platelet count and MPV were determined using an automated hematology analyzer (UniCel DxH 800, Beckman Coulter), and quantitative determination of CRP in human serum was performed via high sensitive immunonephelometry implemented on an automatic analyzer (Beckman Coulter) according to the manufacturer’s instructions.

The measurement of the PCT levels was performed using the ECLIA (electrochemiluminescence immunoassay) sandwich principle method (Cobas e 411, Elecsys BRAHMS PCT test, Roche Diagnostics GmbH, Mannheim, Germany). The measuring range was 0.02–100 ng/mL. Samples in the measurement range were diluted 1:4 with negative human serum. The concentration of the diluted sample was > 1.0 ng/mL. The functional sensitivity was 0.05 ng/mL, the analytical sensitivity was < 0.02 ng/mL, and the detection limit was < 0.02 ng/mL. The levels of PCT were measured to have an intra-assay of coefficient of variation (CV) < 2.7% and an interassay CV of < 5.0%.

### Statistical analyses

Statistical analyses were performed with SPSS 19.0 software (SPSS Inc., Chicago, IL, USA). The distribution of the data was determined by the Shapiro-Wilk test. Continuous variables are expressed as median (min-max), and categorical variables are expressed as frequency and percent. A Pearson Chi-square test was used to determine differences between groups for categorical variables. Continuous variables were compared with the Mann-Whitney U test for two groups. Repeated measures were evaluated with the Friedman test. Dunn’s test was used for the post hoc test after the Friedman test. A receiver operating characteristic (ROC) analysis was constructed to determine the best cut-off value to predict the outcome. The probability was calculated using a logistic regression model, and the estimated probabilities were used in a ROC analysis to calculate the area under curve (AUC) for different models. A *p* value of < 0.05 was considered statistically significant for all tests. At the planning stage of our study, the sepsis group included 240 patients. A preliminary power analysis was performed, and to achieve a 5% type I error probability and 80% prior power with 0.60 effect size, the sample size for control group was determined to be 240 patients. In sepsis group and control group, 36 and 17 of patients were full-term respectively. Because of making a comparison between preterm infants and full-terms were not possible statistically, full-term neonates were excluded from the study. In the study period, 204 preterm newborns who fulfilled the criteria of sepsis group, 223 preterm newborns who fulfilled the criteria of control group included the study.

## Results

A total of 427 premature newborns (207 boys/48.5%, 220 girls/51.5%) were involved in this study. There were 204 newborns in the sepsis group and 223 newborns in the control group. Of the 204 infants with sepsis, 98 (48.0%) were diagnosed with proven sepsis. The remaining 106 infants (52.0%) had clinical sepsis. 98 (48.0%) of the patients were diagnosed with EONS, whereas 106 (52.0%) of the newborns were diagnosed with LONS. A total of 42 septic newborns (20.6%) (34 had proven sepsis and 8 had clinical sepsis) died during the observation period.

The pathogens isolated from the blood were approximately equally divided among Gram-negative (*n* = 50, 51%) and Gram-positive (*n* = 48, 49%) organisms. The two most common Gram-negative pathogens responsible for sepsis were *Escherichia coli* (22/98, 22,4%), and *Klebsiella pneumoniae* (20/98, 20,4%). Extended-spectrum beta-lactamase (ESBL) production was detected in 13 *E. coli*/*K. pneumoniae* isolates. *S. epidermidis* was the most commonly isolated Gram-positive pathogen (32/50, 64%). Methicillin resistance was detected in 36 of 42 (82%) *S. aureus/epidermidis* isolates. In EONS sepsis, Gram-positive bacteria were more common (27/46, 58.7%), whereas Gram-negative pathogens (30/52, 57.7%) were more commonly isolated from the blood of the infants with LONS. The most common pathogens isolated from the infants with EONS were *E.coli* (16/46, 34.8%), methicillin-resistant *S. epidermidis* (14/46, 30.4%) and methicillin-sensitive *S. epidermidis* (8/46, 17.9%). In the infants with LONS, *K. pneumoniae* (20/52, 38.5%) and methicillin-resistant *S. epidermidis* (15/52, 28.8%) were more common detected pathogens.

The differences between the gestational age, birth weight, male gender, and vaginal delivery rate and comparisons of the markers of sepsis are shown in Table 2. CRP 1 (*p* < 0.001), PCT 1 (*p* < 0.001) and MPV 1 (*p* < 0.001) measurements were significantly higher in the infected preterm infants versus the control group. Platelet counts for the initial diagnosis of sepsis were significantly lower in the infected infants (*p* = 0.001) when compared to infants in the control group (Table 2). The differences in the measurements of the platelet, MPV, CRP and PCT levels of the premature sepsis patients are presented in Table 3. The median second-day platelet measurement was significantly lower (*p* = 0.008) in the patients with EONS. The median MPV 1 (*p* < 0.001) and the MPV 2 (*p* = 0.012) levels were significantly higher in LONS group than the neonates with EONS. The median PCT 1 (*p* < 0.001) and PCT 2 measurements (*p* < 0.001), the CRP 1 level (*p* < 0.001), and the MPV 2 (*p* < 0.001) measurement were significantly higher in the proven sepsis group than the neonates with clinical sepsis (Table 3).

**Table 2:** Demographical characteristics and laboratory values for the sepsis and control patients

	Sepsis *n* = 204	Control group *n* = 223	*p*
Gender, male n (%)	92 (45.1)	115 (51.6)	0.181
Vaginal delivery, n (%)	21.0 (10.3)	15.0 (6.7)	0.250
Age of mother (years) med (min-max)	29.0 (16.0–40.0)	29.0 (19.0–43.0)	0.592
Birth weight med (min-max)	1330.0 (550.0–2830.0)	1500.0 (640.0–2960.0)	0.025
Chorioamnionitis, n (%)	21.0 (10.3)	0.0 (0.0)	< 0.001
Early membrane rupture, n (%)	24.0 (11.8)	0.0 (0.0)	< 0.001
Thrombocytopenia, n (%)	71.0 (34.8)	7.0 (3.1)	< 0.001
Mortality, n (%)	42.0 (20.6)	0.0 (0.0)	< 0.001
Platelet 1 (× 10^3^/mm^3^) med (min-max)	225.0 (23–601)	250.0 (93–705)	0.001
CRP 1 mg/L med (min-max)	15.0 (0.0–200.0)	1.5 (0.0–13.0)	< 0.001
PCT 1 ng/mL med (min-max)	10.1 (0.0–200.0)	0.2 (0.0–11.0)	< 0.001
MPV 1 fL med (min-max)	8.4 (5.9–12.1)	7.8 (6.0–11.2)	< 0.001

*CRP* C-reactive protein, *PCT* Procalcitonin, *MPV* Mean platelet volume

**Table 3:** Sepsis patients’ laboratory values

	EONS *n* = 98	LONS *n* = 106	*p*	Proven sepsis *n* = 98	Clinical sepsis *n* = 106	*p*
Platelet 1 × 10^3^/mm^3^ med (min-max)	227.0 (40.0–442.0)	220.0 (23.0–601.0)	0.473	234.0 (23.0–498.0)	221.0 (40.0–601.0)	0.435
Platelet 2 × 10^3^/mm^3^ med (min-max)	192.0 (13.0–649.0)	223.0 (13.0–661.0)	0.008	209.5 (13.0–661.0)	212.5 (27.0–649.0)	0.036
CRP 1 mg/L med (min-max)	10.6 (0.0–200.0)	20.5 (0.0–170.0)	0.113	20.5 (0.6–170.0)	8.25 (10.0–200.0)	0.001
CRP 2 mg/L med (min-max)	3.6 (0.0–203.0)	6.2 (0.0–219.0)	0.075	5.4 (0.0–219.0)	3.8 (0.0–203.0)	0.103
PCT 1 ng/ml med (min-max)	9.6 (0.0–200.0)	10.1 (0.1–109.5)	0.107	18.0 (0.0–200.0)	1.6 0.0–200.0)	< 0.001
PCT 2 ng/ml med (min-max)	2.5 (0.1–200.0)	1.8 (0.1–200.0)	0.131	8.8 (0.1–120.0)	1.2 (0.1–120.0)	< 0.001
MPV 1 fL med (min-max)	8.0 (6.5–11.9)	8.9 (5.9–12.1)	< 0.001	8.5 (6.6–12.1)	8.3 (5.9–11.9)	0.239
MPV 2 fL med (min-max)	8.9 (6.7–11.2)	9.4 (6.3–13.5)	0.012	9.5 (6.7–11.8)	8.7 (6.3–13.5)	< 0.001

*EONS* Early neonatal sepsis, *LONS* Late onset neonatal sepsis, *CRP* C-reactive protein, *PCT* Procalcitonin, *MPV* Mean platelet volume

The optimum cut-off values for CRP, PCT and MPV were identified by drawing ROC curves. The cut-off values are shown in Table 4. For EONS, the cut-off of the CRP 1 was found to be 2.6 mg/L, the sensitivity, specificity, PPV and NPV were 80.6, 83.0, 67.5 and 90.7%, respectively (*p* < 0.001). For the diagnosis of the same group, at a PCT 1 cut-off level of 1.1 ng/mL, the sensitivity, specificity, PPV and NPV were 78.6, 81.2, 64.7 and 89.6%, respectively (*p* < 0.001).The optimum cut-off value of the CRP 1 was 3.6 mg/L for the diagnosis of LONS (*p* < 0.001). The sensitivity, specificity, PPV, and NPV of the CRP 1 cut-off for LONS were 78.3, 87.4, 74.8, and 89.4%, respectively. For the diagnosis of LONS, at a PCT 1 cut-off value of 5.2 ng/mL, the sensitivity, spesificity, PPV and NPV were 58.5, 95.5, 86.1 and 82.9% respectively. For proven sepsis, the cut-off level of the CRP 1 was 7.0 mg/L with 76.5% sensitivity, 98.2% specificity, 94.9% PPV and 90.5% NPV according to the ROC curves (*p* < 0.001). For proven sepsis, at a PCT 1 cut-off level of 1.36 ng/mL, the sensitivity, specificity, PPV and NPV were 90.8, 83.4, 70.6 and 94.4%, respectively (*p* < 0.001). After sepsis diagnosis, the second day cut-off values were shown in the same table (Table 4). The significant CRP 2 cut-off levels of LONS group and proven sepsis group were found to be lower than the initial values. For the patients with LONS, at a cut-off level of 2.4 mg/L for CRP 2, the sensitivity, specificity, PPV and NPV were 69.8, 80.3, 62.7 and 84.8% respectively. For proven sepsis, the cut-off level of CRP was found to be 2.6 mg/L with 70.4% sensitivity, 83.0% specificity, 64.5% PPV and 86.4% NPV (Table 4).

**Table 4:** Cut-off levels for procalcitonin, C-reactive protein and mean platelet volume in preterm neonatal sepsis subgroups

	Cut-off	Sensitivity % (95% CI)	Specificity % (95% CI)	PPV %	NPV%	AUC	*p*
EONS PCT 1 ng/mL	1.1	78.6 (69.1–86.2)	81.2 (75.4–86.1)	64.7	89.6	0.832	< 0.001
EONS PCT 2 ng/mL	0.48	83.7 (74.8–90.4)	67.3 (60.7–73.4)	52.9	90.4	0.801	< 0.001
EONS CRP 1 mg/L	2.6	80.6 (71.4–87.9)	83.0 (77.4–87.6)	67.5	90.7	0.838	< 0.001
EONS CRP 2 mg/L	2.6	59.2 (48.8–69.0)	83.0 (77.4–87.6)	60.4	82.2	0.696	< 0.001
EONS MPV 1 fL	7.9	61.2 (50.8–70.9)	54.3 (47.5–60.9)	37.0	76.1	0.563	0.073
EONS MPV 2 fL	8.2	69.4 (59.3–78.3)	66.4 (59.8–72.5)	47.6	83.1	0.704	< 0.001
LONS PCT 1 ng/mL	5.2	58.5 (48.5–68.0)	95.5 (91.9–97.8)	86.1	82.9	0.820	< 0.001
LONS PCT 2 ng/mL	1.1	60.4 (50.4–69.7)	81.2 (75.4–86.1)	60.4	81.2	0.745	< 0.001
LONS CRP 1 mg/L	3.6	78.3 (69.2–85.7)	87.4 (82.4–91.5)	74.8	89.4	0.856	< 0.001
LONS CRP 2 mg/L	2.4	69.8 (60.1–78.3)	80.3 (74.4–85.3)	62.7	84.8	0.791	< 0.001
LONS MPV 1 fL	8.4	60.4 (50.4–69.7)	72.2 (65.8–78.0)	50.8	79.3	0.689	< 0.001
LONS MPV 2 fL	8.4	75.5 (66.2–83.3)	72.2 (65.8–78.0)	56.3	86.1	0.786	< 0.001
Proven sepsis PCT 1 ng/mL	1.4	90.8 (83.3–95.7)	83.4 (77.9–88.0)	70.6	94.4	0.936	< 0.001
Proven sepsis PCT 2 ng/mL	1.1	75.5 (65.8–83.6)	81.2 (75.4–86.1)	63.8	88.3	0.829	< 0.001
Proven sepsis CRP 1 mg/L	7.0	76.5 (66.9–84.5)	98.2 (95.5–99.5)	94.9	90.5	0.922	< 0.001
Proven sepsis CRP 2 mg/L	2.6	70.4 (60.3–79.2)	83.0 (77.4–87.6)	64.5	86.4	0.771	< 0.001
Proven sepsis MPV 1 fL	7.9	73.5 (63.6–81.9)	54.3 (47.5–60.9)	41.4	82.3	0.653	< 0.001
Proven sepsis MPV 2 fL	8.9	71.4 (61.4–80.1)	82.5 (76.9–87.3)	64.2	86.8	0.811	< 0.001
Clinical sepsis PCT 1 ng/mL	1.2	57.6 (47.6–67.1)	82.1 (76.4–86.9)	60.4	80.3	0.724	< 0.001
Clinical sepsis PCT 2 ng/mL	0.5	67.9 (58.2–76.7)	67.3 (60.7–73.4)	49.7	81.5	0.720	< 0.001
Clinical sepsis CRP 1 mg/L	2.6	73.6 (64.1–81.7)	83.0 (77.4–87.6)	67.2	86.9	0.779	< 0.001
Clinical sepsis CRP 2 mg/L	2.4	59.4 (49.5–68.9)	80.3 (74.4–85.3)	58.9	80.6	0.722	< 0.001
Clinical sepsis MPV 1 fL	9.1	30.2 (21.7–39.9)	88.3 (83.4–92.2)	55.2	72.7	0.034	0.002
Clinical sepsis MPV 2 fL	8.1	72.6 (63.1–80.8)	62.3 (55.6–68.7)	47.8	82.7	0.686	< 0.001

*EONS* Early onset neonatal sepsis, *LONS* Late onset neonatal sepsis, *PPV* Positive predictive value, *NPV* Negative predictive value, *AUC* Area under curve, *PCT* Procalcitonin, *CRP* C-reactive protein, *MPV* Mean platelet volume

**Fig. 1:**
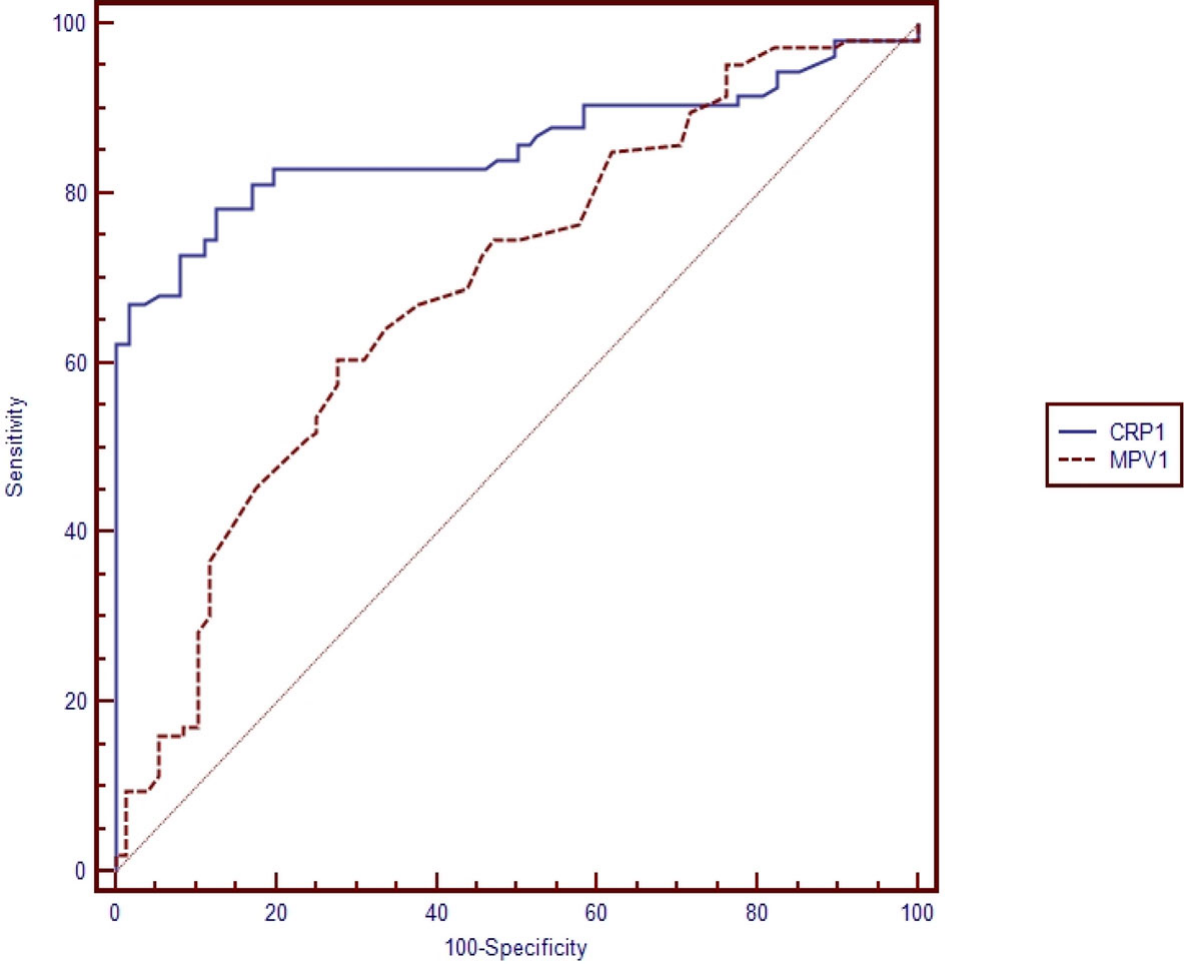
Pairwise comparison of receiver operating characteristic (ROC) curves of C-reactive protein (CRP 1) and mean platelet volume (MPV 1) level on the first day of sepsis diagnosis in late onset preterm neonatal sepsis (LONS)

**Fig. 2:**
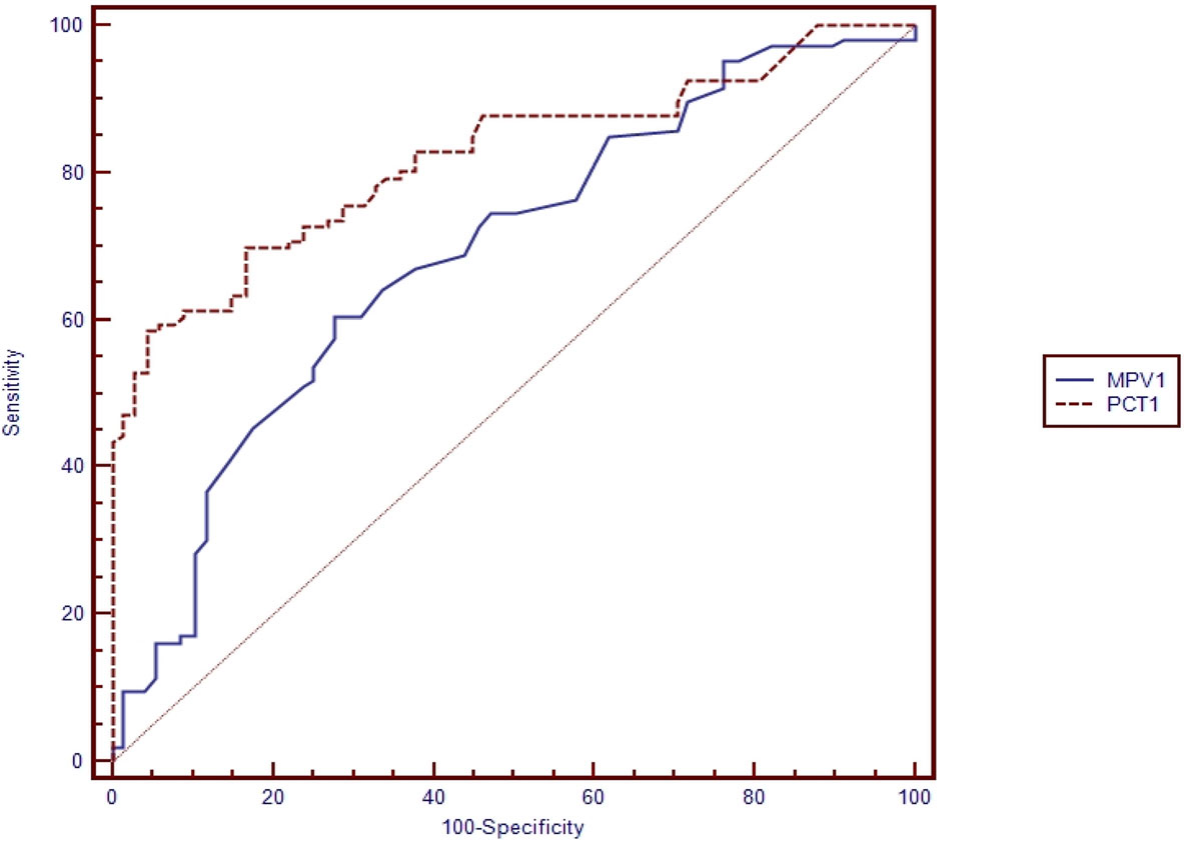
Pairwise comparison of receiver operating characteristic (ROC) curves of mean platelet volume (MPV 1) and procalcitonin (PCT 1) level on the first day of sepsis diagnosis in late onset preterm neonatal sepsis (LONS)

**Fig. 3:**
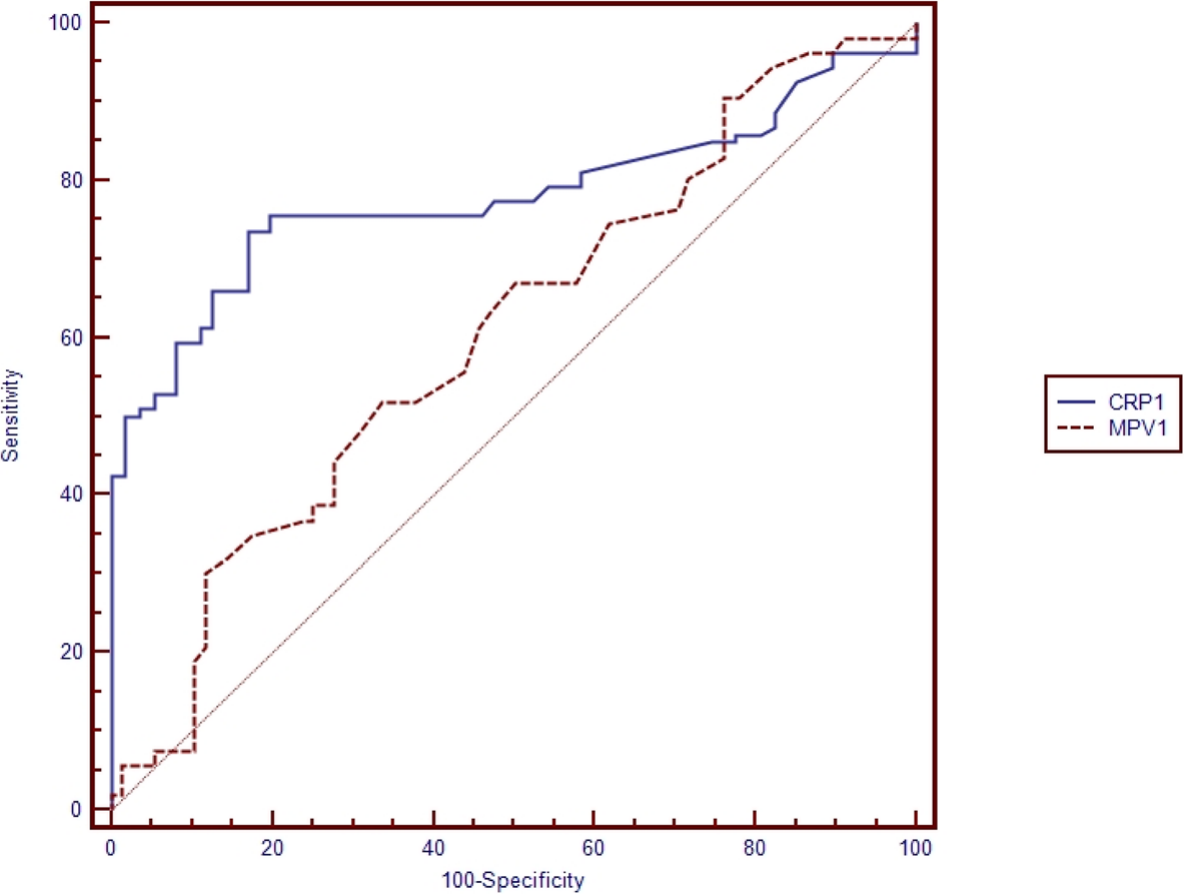
Pairwise comparison of receiver operating characteristic (ROC) curves of C-reactive protein (CRP 1) and mean platelet volume (MPV 1) level on the first day of sepsis diagnosis in clinical preterm sepsis

**Fig. 4:**
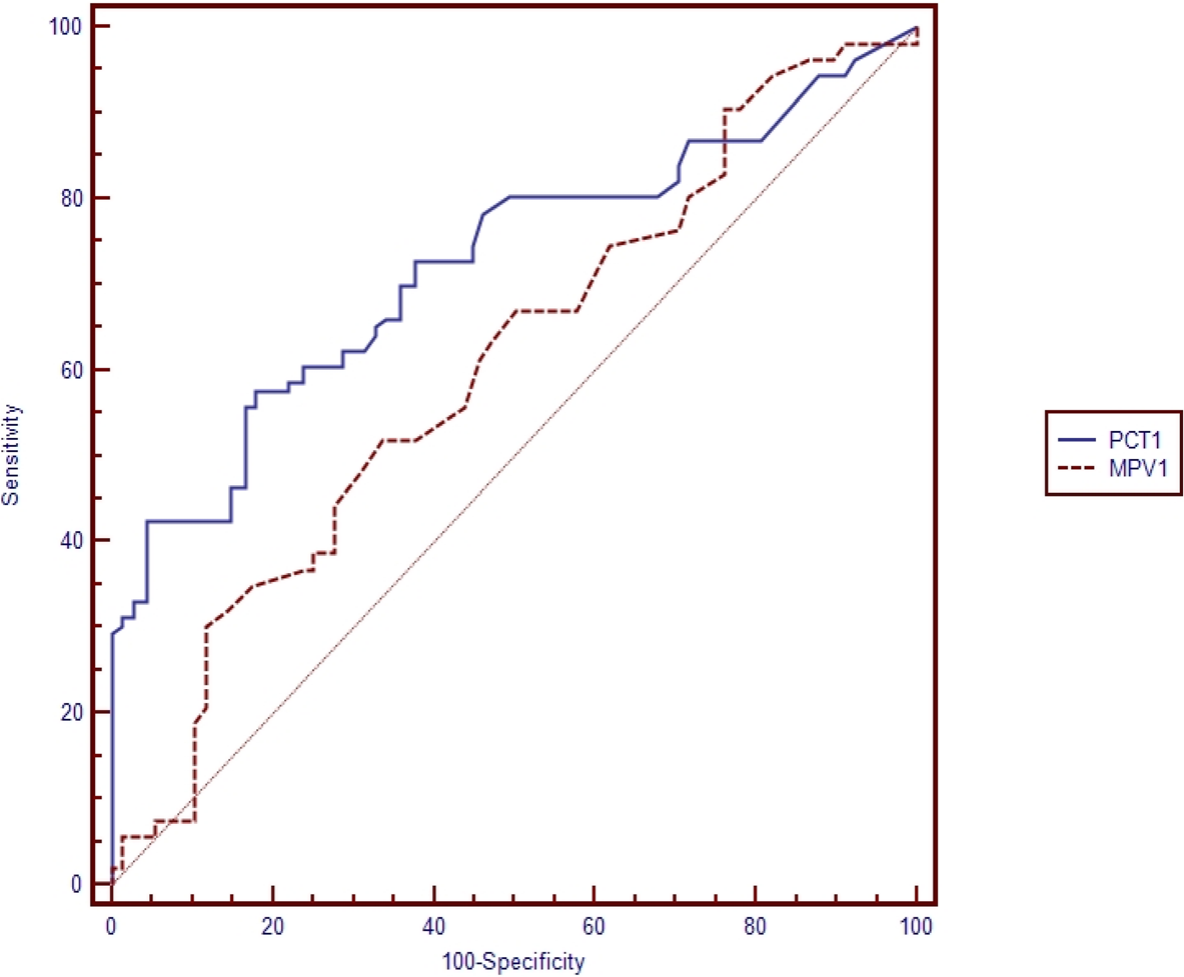
Pairwise comparison of receiver operating characteristic (ROC) curves of mean platelet volume (MPV 1) and procalcitonin (PCT 1) level on the first day of sepsis diagnosis in clinical preterm sepsis

**Fig. 5:**
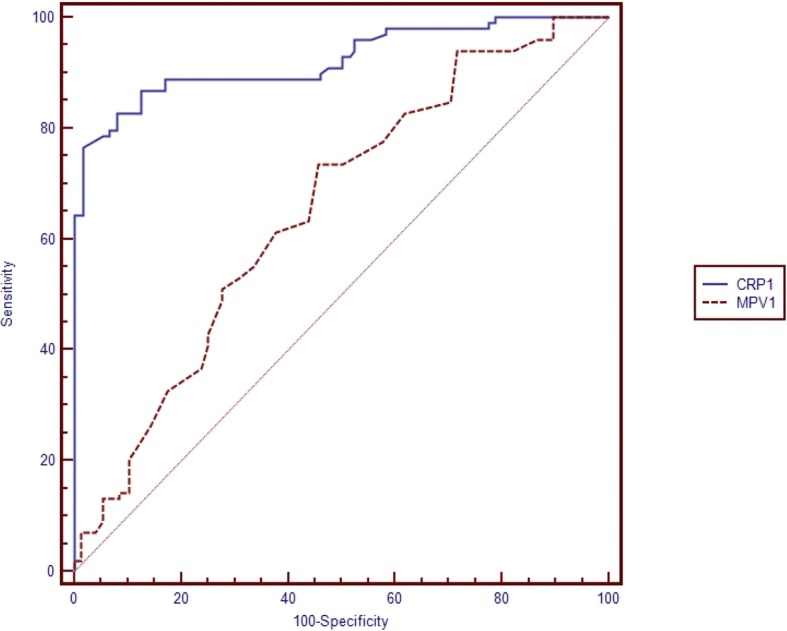
Pairwise comparison of receiver operating characteristic (ROC) curves of C-reactive protein (CRP 1) and mean platelet volume (MPV 1) level on the first day of sepsis diagnosis in proven sepsis

**Fig. 6:**
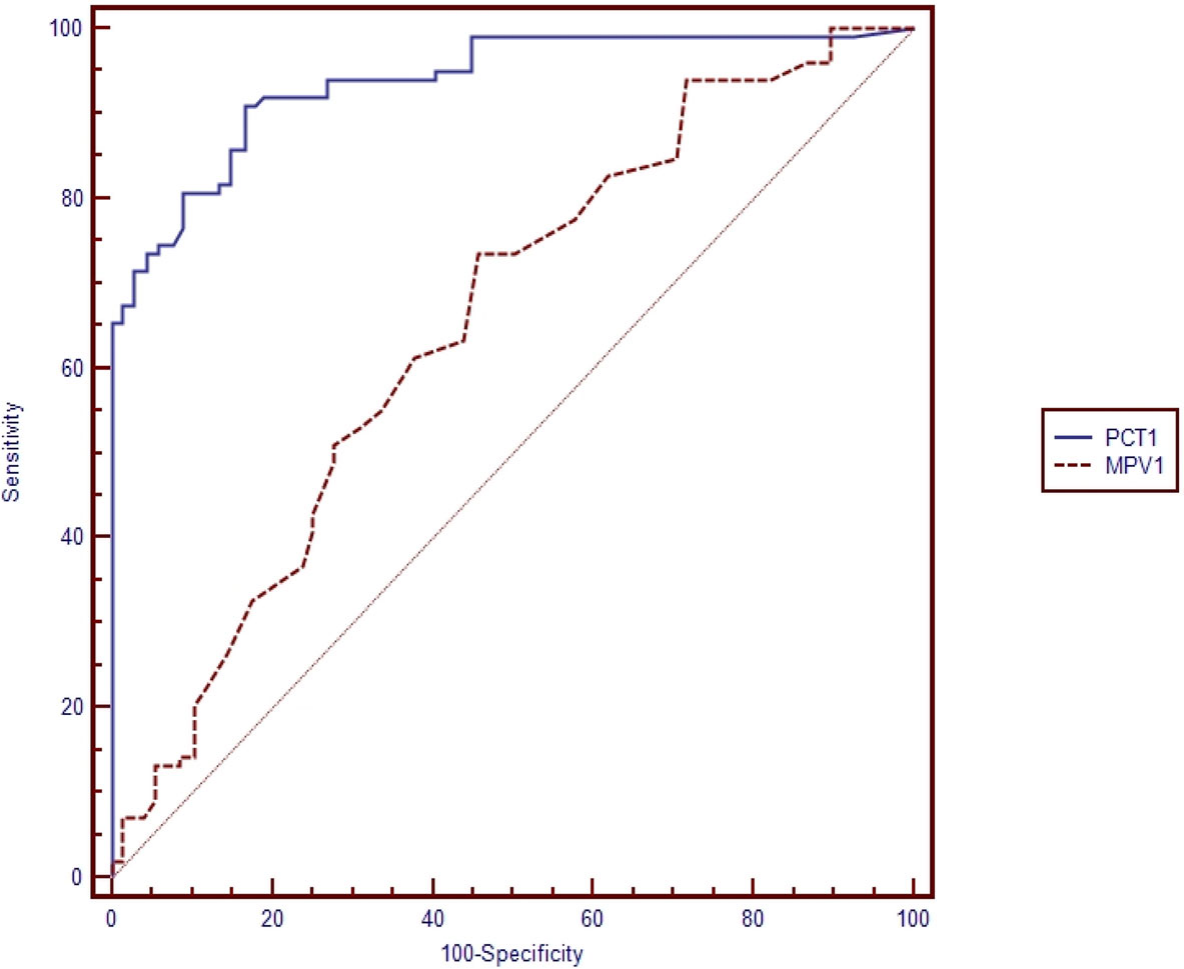
Pairwise comparison of receiver operating characteristic (ROC) curves of mean platelet volume (MPV 1) and procalcitonin (PCT 1) level on the first day of sepsis diagnosis in proven sepsis

Table 5 shows the pairwise comparison of ROC curves of CRP, PCT and MPV for the diagnosis of sepsis subgroups (Table 5). Pairwise comparisons were made if the cut-off level of the study parameter (CRP, PCT, MPV) was found to be statistically significant (Figs. 1, 2, 3, 4, 5 and 6). At the time of suspicion of sepsis, in each sepsis subgroup, other than EONS, the comparison of ROC curves of CRP and MPV and the comparison of ROC curves of PCT and MPV (*p* < 0.001) were found to be statistically significant. Area under curve (AUC) of PCT and AUC of CRP were higher than AUC of MPV in the study sepsis subgroups (Table 5). In each group, no statistically significant difference was found between the comparison of ROC curves of CRP 1 and PCT 1.

**Table 5:** Pairwise comparison of receiving operating characteristic curves in preterm neonatal sepsis subgroups at the time of clinical suspicion of sepsis

	95% CI	*P*
EONS CRP 1 -EONS PCT 1 AUC CRP 1 = 0.838 AUC PCT 1 = 0.832	−0.061-0.074	0.851
LONS CRP 1- LONS PCT 1 AUC CRP 1 = 0.856 AUC PCT 1 = 0.820	−0.027-0.099	0.260
LONS MPV1- LONS CRP 1 AUC MPV 1 = 0.689 AUC CRP 1 = 0.856	0.091–0.243	< 0.001
LONS MPV1- LONS PCT 1 AUC MPV 1 = 0.689 AUC PCT 1 = 0.820	0.055–0.207	0.001
Clinical sepsis CRP 1- Clinical sepsis PCT 1 AUC CRP 1 = 0.779 AUC PCT 1 = 0.724	−0.021-0.131	0.155
Clinical sepsis CRP 1- Clinical sepsis MPV 1 AUC CRP 1 = 0.779 AUC MPV 1 = 0.606	0.086–0.259	< 0.001
Clinical sepsis PCT 1- Clinical sepsis MPV 1 AUC PCT 1 = 0.724 AUC MPV 1 = 0.606	0.033–0.202	0.006
Proven sepsis CRP 1-Proven sepsis PCT 1 AUC CRP 1 = 0.922 AUC PCT 1 = 0.939	−0.034–0.063	0.556
Proven sepsis CRP 1-Proven sepsis MPV 1 AUC CRP 1 = 0.922 AUC MPV 1 = 0.653	0.196–0.343	< 0.001
Proven sepsis MPV 1-Proven sepsis PCT 1 AUC MPV 1 = 0.606 AUC PCT 1 = 0.939	0.212–0.355	< 0.001

*EONS* Early onset neonatal sepsis, *LONS* Late onset neonatal sepsis, *AUC* Area under curve: *CRP* C-reactive protein, *PCT* Procalcitonin, *MPV* Mean platelet volume

The ROC curves of CRP 1 and PCT 1 were combined in order to test whether this improves the diagnostic accuracy. The combinations were found statistically significant for EONS and LONS groups. For patients with EONS, the combination of CRP 1 (> 2.6 mg/L) and PCT 1(> 1.1 ng/mL) had 92.2% sensitivity, 41.9% specificity, 62.1% PPV, and 83.9% NPV. Thus, where sensitivity of the combination was higher, specificity was lower. For the diagnosis of LONS group, the combination of CRP 1 (> 3.6 mg/L) and PCT 1(> 5.2 ng/mL) had sensitivity, specificity, PPV, and NPV of 74.1, 80.0, 83.3, and 69.6%, respectively. According to the ROC curves, whereas the sensitivity of this combination was higher than the sensitivity of PCT 1 alone, the specificity was lower than PCT 1. The sensitivity and the specificity of the combination of ROC curves were similar to the sensitivity and the specificity of CRP 1.

## Discussion

Sepsis is a complex syndrome with significant morbidity and mortality. Despite recent advances in diagnosis, establishing definitive diagnostic criteria may be difficult. Developmental differences between children and adults lead to distinct variations in the epidemiology, pathophysiology, diagnosis and management in children compared with adults [15]. In addition to the clinical signs of sepsis, many laboratory biomarkers are under investigation to enable a rapid diagnosis of sepsis. Elevated PCT levels are nearly pathognomonic for sepsis and may be used to guide management, however the cut-off levels of PCT may vary and should not be used alone in the diagnosis of neonatal sepsis [16]. MPV values increase as a result of increased platelet production and/or increased platelet destruction in sepsis [17]. Although a substantial number of studies have focused on the relationship between sepsis and thrombocytopenia, few studies have investigated MPV kinetics.

CRP is one of the most studied and utilized laboratory markers for newborn sepsis. Because of the delay in synthesis, it may be low early in infection [18]. Moreover, non-infectious factors may influence CRP kinetics, for example; complications at delivery have been associated with non-specific elevations of CRP in the early perinatal period [18, 19]. In previous studies, the range of CRP sensitivity and specificity has been reported as 35–94% and 60–96%, respectively [19]. In a recently published study, CRP measurements were compared between EONS and LONS. The CRP levels of EONS were significantly lower than the levels of LONS. Moreover, CRP had 75% sensitivity and 76.3% specificity for proven sepsis with a cut-off of 0.16 mg/dL [2]. From previously published studies CRP was reported to have low sensitivity during the first hours of sepsis [18]. In our study, the median CRP 1 level was significantly higher in the patients with LONS than the patients with EONS. The optimum cut-off value in the diagnosis of LONS was 3.6 mg/L for CRP 1 in this study. The sensitivity, specificity, PPV, and NPV of the CRP 1 cut-off for the diagnosis of LONS were 78.3, 87.4, 74.8, and 89.4%, respectively. In EONS, we found the cut-off level of CRP 1 for the diagnosis of sepsis to be 2.6 mg/L with a 80.6% sensitivity, 83.0% specificity, 67.5% PPV and 90.7% NPV. Chiesa et al. investigated the reference interval of CRP in preterm newborns with EONS. They found the predicted CRP to be 0.1 mg/L at birth and the level increased to 1.7 at 27–36 h. Their birth cut-off level of CRP was lower than our cut-off level [5]. In another published study, the CRP and IL-6 cut-off levels were investigated in newborns. No significant difference was reported in the CRP levels between the proven and clinical sepsis groups; however, the authors identified a significant difference between the septic newborns and the control group. The cut-offs of CRP were determined to be 0.58 mg/dL and 0.48 mg/dL for proven sepsis and all sepsis, respectively. For proven sepsis, the sensitivity, specificity, PPV and NPV were 71, 97, 99 and 49%, respectively. The authors noted that the combination of CRP and IL6 may be more helpful with higher sensitivity and specificity [20]. In our study, it was striking that the median CRP 2 levels for all types of sepsis were found to be lower than the initial values. We found significant cut-offs of CRP 2 levels to be lower than the initial values in LONS and proven sepsis groups. We think it may be due to the response to antibiotics that were given promptly when the sepsis diagnosis was established. We also investigated the combination of CRP 1 and PCT 1 for the diagnosis of EONS and LONS. Acoording to the ROC curves, the sensitivity of this combination was found to be higher than CRP 1 or PCT 1 alone in EONS. In a recently published study, salivary CRP and MPV were investigated for the diagnosis of septic neonates and they found significant difference of CRP levels between septic neonates and controls. At a cut-off of 3.48 ng/L, salivary CRP showed high specifity and sensitivity [21]. In our study, we determined the cut-off of CRP 1 to be 7.0 mg/L for proven sepsis. At this cut-off, the sensitivity, specificity, PPV, and NPV were 76.5, 98.2, 94.9 and 90.5%, respectively. For the diagnosis of clinical sepsis, at the CRP 1 cut-off of 2.6 mg/L, the sensitivity, specificity, PPV and NPV were 73.6, 83.0, 67.2 and 86.9% respectively. For the diagnosis of proven sepsis, we found the cut-off of CRP 2 to be 2.6 mg/L. This second day CRP cut-off was lower than the the cut-off of first day CRP. The specifity, PPV and NPV of this cut-off were also lower than the first day values.

Patrick et al. evaluated 156 newborns and demonstrated that MPV measurements were considerably higher in patients with bacteremia than newborns without infection. The authors reported the sensitivity and specificity of MPV for the diagnosis of sepsis to be 42 and 95%, respectively [22]. Oncel et al. indicated significantly higher MPV and CRP levels in newborns with sepsis than healthy controls [23]. Cekmez et al. investigated the relationship of MPV between the various diseases of newborns other than sepsis. The authors identified high MPV levels within the first hours of these diseases; however, their data showed that higher MPV values were not associated with the development of sepsis [24]. We identified significantly higher MPV levels for the first day of diagnosis in septic premature newborns than non-infectious premature controls. In our study, the median MPV 1 and MPV 2 levels were significantly higher in the LONS group than the premature newborns with EONS. We did not find any statistically significant difference in MPV level between patients with proven and clinical sepsis. But in proven sepsis group, we determined significantly higher MPV 2 level than the premature newborns in clinical sepsis group.

Currently, PCT appears to be a specific alternative marker to CRP in the rapid diagnosis of sepsis. The cut-off levels of PCT in newborns with sepsis have been investigated in the literature. Falsely increased PCT concentrations have been reported for hospitalized newborns as a result of non-infectious, critical diseases [25–27]. Moreover, it has been reported that the PCT levels may be normal in severely infected newborns [26–28]. The clinical signs and symptoms of sepsis are silent; low or normal PCT levels may make the diagnosis of sepsis complex. Altunhan et al. compared the PCT levels between septic neonates and non-infectious patients for the diagnosis of EONS. They did not identify a difference between the levels at birth; however, at 24 h of age, the PCT levels were significantly higher in the newborns with suspected sepsis. At 24 h of age, they used a cut-off value of 5.38 ng/mL and determined that the specificity, sensitivity, PPV and NPV were increased when compared with the use of the cut-off value of 0.59 ng/mL at birth [29]. We did not identify a significant difference between the levels of PCT in EONS and LONS. In our study, the median PCT levels at birth and at 24 h of age were significantly higher in patients with proven sepsis. We also determined significant cut-off values for PCT 1 and PCT 2 levels for the diagnosis of proven sepsis. The PCT 1 and PCT 2 cut-off values were found to be 1.4 and 1.1 ng/mL, respectively (sensitivity: 90.8%/75.5%, specificity: 83.4%/81.2%, PPV: 70.6%/63.8% and NPV: 94.4%/88.3%). At a PCT 1 cut-off value of 1.1 ng/mL, the sensitivity, specificity, PPV and NPV were 78.6, 81.2, 64.7 and 89.6% respectively. In a recently published systematic review, it was reported that the evaluation of the clinical usefulness of PCT in ruling in or out neonatal sepsis, in particular EONS, was dependent on the study consistency [25]. In literature we could not find a study included the pairwise comparison of the performance of the parameters of CRP, MPV and PCT in premature neonatal sepsis patients. In this study, the performance of CRP 1 and PCT 1 were found to be significantly higher than the performance of MPV 1 in each sepsis sub-group other than EONS. We did not find a significant cut-off level of birth MPV for the diagnosis of EONS, so pairwise comparisons did not include this parameter. The performances of CRP 1 and PCT 1 did not differ in each sub-group. In the literature, it was reported that serial PCT measurements, not only at the time of clinical suspicion of sepsis but also after 24 h, may be helpful in differentiating between sepsis and noninfectious conditions [28]. In septic neonates who had PCT levels higher than 0.59 ng/mL at birth, the authors determined that these levels subsequently further increased, whereas no significant elevation was identified in the non-infectious group. Although it was reported that increasing PCT levels were valuable for sepsis diagnosis, we did not find a similar elevation in PCT levels [28]. Moreover, we found significantly decrease in PCT levels from the first day values in sepsis groups. In a recently published multicentre study, it was reported that PCT-guided follow up the neonates resulted in a significant reduction in therapy duration. Neonates with EONS were categorized free of PCT values in their study. The normal ranges of PCT were determined according to the birth hours. For the first hours of birth, they accepted the normal ranges of PCT to be 0–0.5 ng/mL. The accepted normal upper range increased to 10 ng/mL in the 36th hours of birth [16].

Our study had some limitations for these measurements. It was not prospective so we could not measure these parameters in the following hours and days of sepsis diagnosis. As we have mentioned in the Methods section, the time interval of blood sampling for these measurements was a part of neonatal sepsis protocol in our hospital. And we could not compare the laboratory parameters between the preterm and full-term neonates, because our study population did not include full-term patients.

In our study, in the proven sepsis group, the isolated pathogens from the blood were approximately equally divided among Gram-negative (*n* = 50, 51%) and Gram-positive (*n* = 48, 49.0%) organisms. The two most common Gram-negative pathogens responsible from sepsis were *Escherichia coli* (22/98, 22.4%), and *Klebsiella pneumoniae* (*n* = 20/98, 20.4%). ESBL production was detected in 13 *E. coli*/*K. pneumoniae* isolates. Methicillin resistance was detected in 82% *S. aureus/epidermidis* isolates. In EONS sepsis, Gram-positive bacteria were more common (27/46, 58.7%), whereas Gram-negative pathogens (30/52, 57.7%) were more commonly isolated from the blood of infants with LONS. Celik et al. determined that Gram-positive bacteria were more common among infants from both EONS and LONS groups [2]. In another published study, it was reported that among the blood culture results of 401 neonatal infants, the most isolated microorganisms were *S. aureus/epidermidis* and Group B Streptococci (GBS) [30]. In our study, GBS did not appear more prominently in the culture positive EONS group. We think it may be due to reduction in GBS infections, owing to the use of intra-partum antibiotic prophylaxis. In literature it was reported that the incidence of EONS due to GBS was decreased because of the same reason [31]. In our control group, no deaths were recorded, whereas 20.6% of premature infants died in the sepsis group. This finding was similar to the literature [28]. *S. epidermidis* was the most commonly isolated Gram-positive pathogen. Although this microorganism in culture may suggest contamination, from the literature it was known that it can be pathogenic in neonates with indwelling vascular catheters or other devices [32]. Our study patients were all preterm and most of them had vascular catheters and other invasive devices.

## Conclusions

We determined that the PCT and CRP levels and the MPV measurements are significantly higher in the preterm patients with sepsis. The cut-off levels of CRP and PCT may differ in these subgroups. Furthermore the performances of these tests were found to be different in the diagnosis of premature sepsis subgroups. Although no significantly difference was found between the performances of CRP and PCT measurement in sepsis subgroups, we found that PCT and CRP had higher performances than MPV for the first 24 h of diagnosis of preterm neonatal sepsis.

## Abbreviations

AUC: Area under curve
CI: Confidence interval
CLSI: Clinical and laboratory standarts institude
CRP: C-reactive protein
*E. coli*: *Escherichia coli*
ECLIA: Electrochemiluminescence immunoassay
EONS: Early-onset neonatal sepsis
ESBL: Extended-spectrum beta lactamase
*K. pneumonia*: *Klebsiella pneumonia*
LONS: Late-onset neonatal sepsis
MPV: Mean platelet volume
NPV: Negative predictive value
PCT: Procalcitonin
PPV: Positive predictive value
ROC: Receiver operating characteristic
*S. aureus*: *Staphylococcus aureus*
*S. epidermidis*: *Staphylococcus epidermidis*
